# Biopsy Techniques for Musculoskeletal Tumors: Basic Principles and Specialized Techniques

**DOI:** 10.3390/curroncol31020067

**Published:** 2024-02-05

**Authors:** Andreas F. Mavrogenis, Pavlos Altsitzioglou, Shinji Tsukamoto, Costantino Errani

**Affiliations:** 1 First Department of Orthopaedics, School of Medicine, National and Kapodistrian University of Athens, 1 Rimini, 157 72 Athens, Greece; pavlosaltsi@gmail.com; 2 Department of Orthopaedic Surgery, Nara Medical University, 840 Shijo-cho, Kashihara 634-8521, Japan; sh104@naramed-u.ac.jp; 3 Department of Orthopaedic Oncology, IRCCS Istituto Ortopedico Rizzoli, Via Pupilli 1, 40136 Bologna, Italy; costantino.errani@ior.it

**Keywords:** biopsy, percutaneous, incisional, liquid

## Abstract

Biopsy is a pivotal component in the diagnostic process of bone and soft tissue tumors. The objective is to obtain adequate tissue without compromising local tumor dissemination and the patient’s survival. This review explores contemporary principles and practices in musculoskeletal biopsies, emphasizing the critical role of diagnostic accuracy while also delving into the evolving landscape of liquid biopsies as a promising alternative in the field. A thorough literature search was done in PubMed and Google Scholar as well as in physical books in libraries to summarize the available biopsy techniques for musculoskeletal tumors, discuss the available methods, risk factors, and complications, and to emphasize the challenges related to biopsies in oncology. Research articles that studied the basic principles and specialized techniques of biopsy techniques in tumor patients were deemed eligible. Their advantages and disadvantages, technical and pathophysiological mechanisms, and possible risks and complications were reviewed, summarized, and discussed. An inadequately executed biopsy may hinder diagnosis and subsequently impact treatment outcomes. All lesions should be approached with a presumption of malignancy until proven otherwise. Liquid biopsies have emerged as a potent non-invasive tool for analyzing tumor phenotype, progression, and drug resistance and guiding treatment decisions in bone sarcomas and metastases. Despite advancements, several barriers remain in biopsies, including challenges related to costs, scalability, reproducibility, and isolation methods. It is paramount that orthopedic oncologists work together with radiologists and pathologists to enhance diagnosis, patient outcomes, and healthcare costs.

## 1. Introduction

The accurate identification of musculoskeletal tumors is paramount for effective patient treatment and care; the biopsy procedure is a pivotal component in the staging and diagnostic process of the patients with musculoskeletal tumors. Omission of the biopsy may be justified only in instances when there is clear clinical and radiographic evidence of benign lesions, such as chondroma, osteochondroma, osteoid osteoma, simple bone cysts, fibrous dysplasia, or non-ossifying fibroma [1,2]. The primary goal of biopsy is to obtain tissue samples for diagnostic purposes while minimizing patient health risks, reducing the potential for tumor contamination, and safeguarding future treatment options [1]. An inadequately executed biopsy may hinder precise diagnosis and subsequently impact treatment outcomes adversely [2]. A study conducted by Mankin et al. [3], involving 597 individuals who underwent biopsy procedures for bone and soft tissue sarcomas, revealed a diagnostic error of 13.5% and a complication incidence of 15.9%; unnecessary amputations were observed in 3% of their patients with a higher frequency in cases where the biopsy was performed in a referral institution rather than an oncology facility. As a general guideline, it is advisable to approach all lesions with a presumption of malignancy until proven otherwise, suggesting a deferment of the biopsy until comprehensive imaging studies have been conducted [4].

Biopsy is a compromise between representative tissue sampling and avoidance of tissue contamination. Various biopsy techniques, including closed/percutaneous (imaging-guided or free hand; fine-needle aspiration, core needle) and open (incisional or excisional) biopsies, aim to provide representative tissue sample while minimizing complications [5]. The incidence of complications for closed biopsies ranges from 0 to 10%, compared to up to 16% for open biopsies [4]. The primary complications associated with the biopsy approach are hemorrhage, nerve apraxia, and infection [4].

Currently, liquid biopsies have emerged as a potent non-invasive tool for analyzing tumor phenotype, progression, and drug resistance [6]. Particularly in the field of bone oncology, where standard biopsies can be both painful and hazardous, liquid biopsies hold substantial promise [7]. Liquid biopsy methods, such as the analysis of circulating tumor cells (CTCs), cell-free circulating tumor DNA (ctDNA), and extracellular vesicles (EVs), have advanced considerably in recent years. They offer opportunities for improving diagnosis, prognosis, evaluating therapy resistance, and guiding treatment decisions in primary bone sarcomas such as osteosarcoma and Ewing’s sarcoma, as well as secondary bone tumors such as breast, prostate, and lung cancer-induced bone metastases [8].

Despite advancements in biopsy techniques, several barriers remain, including challenges related to costs, scalability, reproducibility, and isolation methods, hindering the broader adoption of biopsies in bone oncology. This review article aims to explore contemporary principles and practices in biopsy techniques in musculoskeletal tumors with emphasis on the critical role of diagnostic accuracy and the evolving landscape of liquid biopsies as a promising alternative in the field.

## 2. Traditional Biopsy Techniques

The principles of biopsy techniques for musculoskeletal tumors remain consistent across different techniques (Table 1). The diagnosis of bone and soft tissue tumors often necessitates the collection of numerous samples due to their inherent heterogeneity. This method does not facilitate the metastatic spread of tumor cells, but it may lead to local dissemination and hence heighten the chance of local recurrence [2,4]. Based on this rationale, it is necessary to hypothesize that the biopsy tract might potentially be compromised, hence necessitating its resection during the ultimate surgical procedure for resection of a sarcoma. Hence, it is essential that the biopsy tract, either closed or open, should be conducted at the predetermined surgical incision location to ensure its inclusion within the definite surgical specimen. The most efficient path to reach the lesion may not always be the shortest distance [4]. Importantly, the biopsy tract should not breach more than one anatomical compartment and remains far from the neurovascular bundle [2].

### 2.1. Fine-Needle Aspiration Biopsy

Fine-needle aspiration biopsy has a limited, if no role at all, in musculoskeletal biopsy. The prevalence of false negatives is substantial; even in cases that the diagnosis yields good results, it is not always possible to achieve a high level of precision [1,7]. One primary constraint is the inability to assess tissue architecture, and moreover, cytologic samples may not always be sufficient for supplementary analyses such as cytogenetic, molecular, or immunohistochemical studies. One benefit of this treatment is its comparatively low topical trauma and incidence of complications, as well as the relatively low cost and morbidity [1]. The technique may be used for both local and distant recurrence scenarios, whereby the cytology results can be juxtaposed with the previous histology specimens [7].

### 2.2. Core-Needle Biopsy

**Figure 1:**
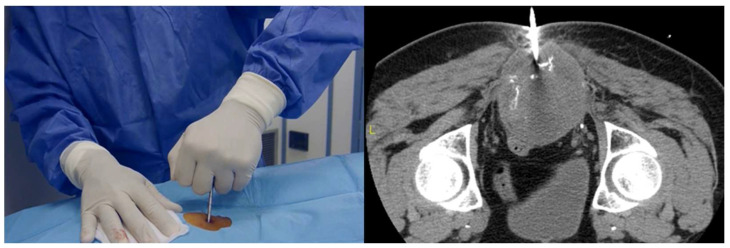
CT-guided trocar needle biopsy for a bone lesion of the sacrum. The trocar is advanced to the tumor through a cannula of 8G (4 mm). Histology showed a giant cell tumor of the bone.

Core-needle biopsy is associated with a low rate of false negative results. The structural integrity of the biopsy tissue is maintained, enabling the potential for histological diagnosis, tumor grading, as well as immunohistochemical or molecular studies. One of the benefits associated with this approach is the reduced risk of local tumor contamination [8]. Additionally, this method is considered less intrusive, thus enhancing its appeal from the patients’ perspective [8]. The use of ultrasonography or CT guidance (Figure 1) is currently considered the gold standard to enhance the accuracy of percutaneous biopsies [1,9,10,11]. Imaging-guided biopsies are very safe when performed by appropriately trained and experienced radiologists and guided by orthopedic oncologist surgeons; exceptionally, several complications have been reported, including pneumothorax, hemorrhage, air embolism, and tumor seeding [1,8,9,10,11,12]. If there is a lack of sufficient tissue, or discrepancy with imaging and the clinical diagnosis, a guided biopsy can be repeated, or an open biopsy should be performed [12].

### 2.3. Incisional Biopsy

**Figure 2:**
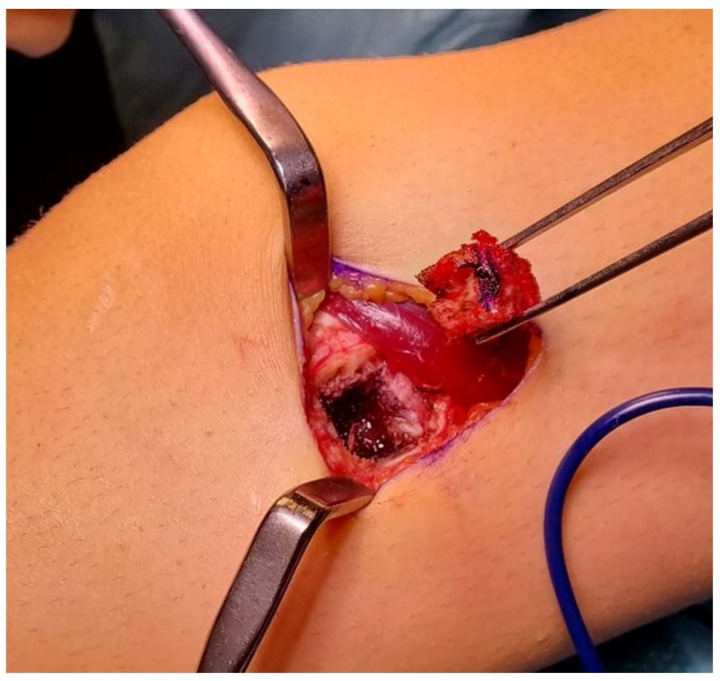
Incisional biopsy for a lesion of the proximal humerus. Histology showed a conventional central chondrosarcoma.

In cases where a core biopsy yielded insufficient results, an open biopsy should be considered [13]. It may be used in conjunction with frozen section analysis to verify the acquisition of diagnostic material, and, in the event of a benign diagnosis, determine the need of an excision. The biopsy incision should be put along the incision line that will be used for the subsequent surgical excision. The use of the smallest incision that is consistent with acquiring a sufficient specimen is obligatory (Figure 2). Transverse incisions are not recommended due to the need of a broader removal of soft tissue during the ultimate surgical procedure [2]. Complete hemostasis is of utmost importance in order to prevent the formation of a hematoma and minimize the potential danger of local tumor dissemination inside the hematoma. The presence of a hematoma in the vicinity of a tumor should be regarded as being contaminated, hence rendering limb salvage operations unfeasible [2]. In the event that a tourniquet is used, it is imperative that it will be released prior to wound closure for careful hemostasis. Typically, the use of suction drains is seldom necessary; in exceptional circumstances when its implementation becomes necessary because of inadequate hemostasis or expected hemorrhage, such as in patients receiving anticoagulation medications, the drains should be positioned in line with the skin incision, approximately 1 cm away [2]. The drain sinus is regarded as contaminated and must be removed together with the surgical specimen and the biopsy tract [2].

Several drawbacks relate to incisional biopsies, such as the potential for spilling of tumor cells and issues related to wound healing. The occurrence of wound problems and the improper positioning of the incision might potentially hinder the effectiveness of local therapy [14]. The performance of an open surgical biopsy has the potential to increase the size of the surgical specimen and adversely affect the functionality of the limb [15].

## 3. Characteristics and Considerations of Biopsy Samples

### 3.1. Bone Specimens

**Figure 3:**
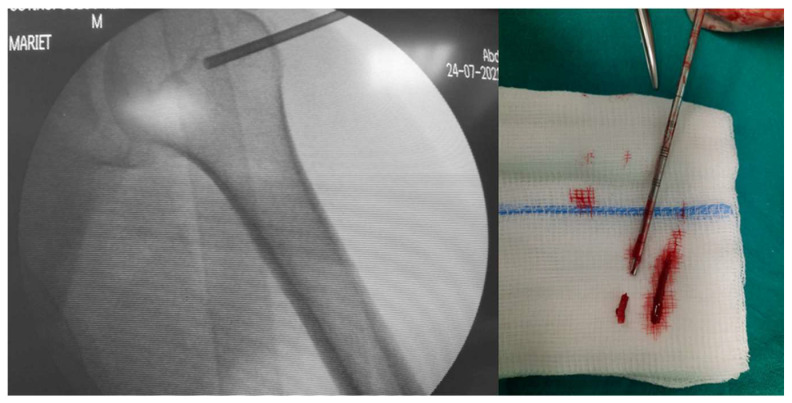
Fluoroscopy-guided trocar needle biopsy for a bone lesion of the humeral head. Histology showed a giant cell tumor of the bone.

The specimens are collected from the outside edges of the tumor, since core necrosis is often seen in bone tumors [5]. In general, it is better to consider the extraosseous component of a presumptive malignant bone tumor as being more representative of the tumor. Fluoroscopy (Figure 3) or CT guidance is recommended. Breaching the cortex of bone increases the likelihood of a pathological fracture and is advised only in cases when there is no tumor expansion beyond the bone. In these cases, it is recommended to perform a cortical window and approach the tumor. The shape of the cortical window may be an important predictor for a pathological fracture. According to the findings of Clark et al. [16], an elongated hole with rounded ends provides a higher level of residual strength in the bone when compared to rectangular-shaped cortical windows with either square or rounded corners. Furthermore, the same authors observed that augmenting the width of the aperture results in a noteworthy decrease in bone strength; however, extending its length does not have a similar effect.

There is no significant difference in diagnostic accuracy between core-needle and open biopsy for bone tumors [5,6,11,17]. In a retrospective study by Pohlig et al. [5], a comparison was made between a closed (core-needle) biopsy and an open biopsy in 48 patients with bone tumors. The core-needle biopsy showed a diagnostic accuracy rate of 100%, whereas the incisional biopsy yielded a diagnostic accuracy rate of 93.3%, without, however, a statistically significant difference between the two approaches.

**Figure 4:**
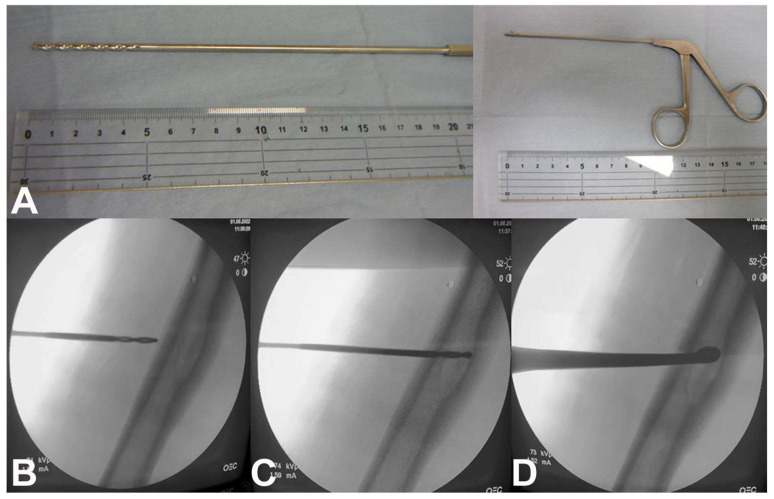
(**A**) A 3.5 mm drill (left) and micro-rongeur (right) used for a fluoroscopy-guided percutaneous biopsy. Fluoroscopy-guided percutaneous biopsy of a femoral diaphysis lesion using a 3.5 mm drill to (**B**) locate and (**C**) drill the cortex and to (**D**) obtain an adequate sample with a curette.

In 14 patients with a bone lesion completely filled with fluid-fluid, a percutaneous needle biopsy was diagnostic in five patients (36%) and non-diagnostic in nine patients (64%) [18]. In addition, surgical curettage/resection in 52 patients was diagnostic in 50 patients (96%) and non-diagnostic in two patients (4%) [18]. These results suggest that lesions with fluid-fluid levels on MR are less likely to be diagnosed correctly through a percutaneous needle biopsy than surgical curettage/resection, likely due to insufficient diagnostic cellular material obtained through a needle biopsy. To avoid this, a small hole can be performed with a 3.5 mm drill through which a rongeur or curette can be inserted to shave off the tumor located in the interior wall of the cavity under fluoroscopy in order to obtain a sufficient sample (Figure 4A–D).

### 3.2. Soft Tissue Specimens

Not all soft tissue lesions require an intervention or a diagnostic biopsy. There are situations in which the clinical and imaging characteristics are sufficiently characteristic of a benign entity such as lipoma, hemangioma, and neurofibroma, a pseudotumor such as ganglions and popliteal cysts, myositis ossificans, and tenosynovial giant cell tumor; in these cases, a biopsy may not be necessary [19]. A biopsy is recommended in cases when a soft tissue lesion exhibits biological activity or tissue growth, in soft tissue masses larger than 3–4 cm in maximal diameter, especially if deep-seated (under the fascia), and in those that cannot be excised with tumor-free (microscopically negative) surgical margins [20]. The biopsy tract (needle or incision) should be positioned exactly above the tumor, that is, at the location where the lesion is closest to the surface. It is important to refrain from elevating flaps or causing any disruption to tissue planes that are superficial to the tumor [21].

The biopsy tissue should be obtained from the outside edges of the lesion with inclusion of the tumor capsule, that is, the reactive zone that by definition contains living tumor cells. It is common to find necrotic and non-diagnostic tissue inside the tumor. Typically, a minimum of three core-needle tumor tissue samples should be obtained [4,20]. The challenge in histological diagnosis is often seen in neoplasms with myxoid and round cell features [20]. The complication rate of an open biopsy for soft tissue tumors may reach up to 16%, including issues such as hematoma formation, tumor dissemination, and wound-related complications that may impede subsequent therapeutic interventions [1,22,23,24].

Fine-needle aspiration biopsy for soft tissue tumors exhibits a broad spectrum of sensitivity (range, 86% to 100%), specificity (range, 36% to 100%), and diagnostic accuracy (range, 21.9% to 98%); it has been used for the purpose of documenting metastases and local recurrences, particularly when previous samples are accessible for comparative histological analysis [24]. However, it lacks the ability to accurately subtype sarcomas [1,24]. In a retrospective study, Ng et al. [24] investigated the diagnostic accuracy of 432 fine-needle aspiration biopsies conducted on soft tissue lesions in the extremities. In 8.1% of the cases, it was stated that the type of the lesion was uncertain or the sample was insufficient. The accuracy rates for subtyping and grading malignant lesions were found to be 77.2% and 95.2%, respectively. According to the cited source, 25% of the patients needed an additional biopsy procedure for optimal diagnosis prior to receiving a conclusive treatment [24].

**Figure 5:**
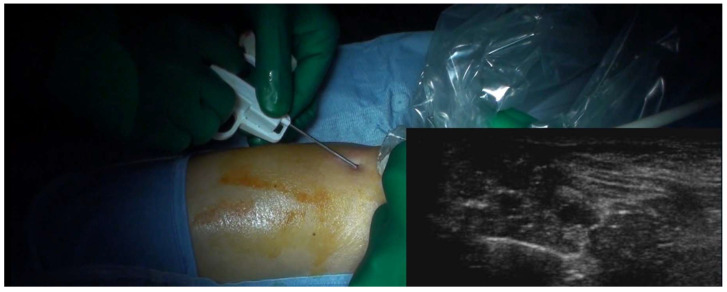
Ultrasonography-guided Tru-cut needle biopsy for a soft-tissue lesion of the popliteal fossa. The needle is semi-automatic and has a size of 16G and a penetration depth of 22 mm. Histology showed a synovial sarcoma.

Percutaneous (core-needle) biopsy has emerged as a viable substitute for fine-needle aspiration biopsy for soft tissue lesions, with enhanced sensitivity (range, 81.8% to 100%), specificity (range, 91% to 100%) and diagnostic accuracy (range, 72.7% to 100%) in determining histologic type and grade of the tumors, and a low incidence of complications (range, 0.1% to 1.1%) [1]. Interestingly, while there is a significant body of research on the diagnostic yield of various biopsy techniques, there are only two studies that have directly examined the accuracy of biopsy techniques in the context of soft tissue tumors [1,7]. In a study by Yang and Damron [7], a comparison was made between fine-needle aspiration and core-needle biopsy for soft tissue lesions; the results showed a higher level of accuracy of core-needle biopsy compared to that of fine-needle aspiration (83% vs. 64%). In a prospective study of Kasraeian et al. [1], the authors used a sequential approach, beginning with a fine-needle aspiration, followed by a core-needle biopsy, and concluding with an incisional biopsy of the same soft-tissue lesions in 57 patients. The incisional biopsy showed complete accuracy in all cases, compared to lower overall accuracy for fine-needle aspiration (75.4%) and core-needle biopsy (80.7%). Based on their results, these authors propose the use of incisional biopsy as a diagnostic approach for soft tissue tumors [1]. Ultrasonography-guided percutaneous biopsies of soft tissue lesions exhibit a notable level of accuracy (Figure 5) [4]. The use of real-time multiplanar viewing of the needle offers a strategy that ensures safety by providing visual representation of essential structures. Additionally, ultrasonography guidance allows for selective sampling of specific regions inside the tumor, therefore avoiding areas that are cystic or necrotic [4].

## 4. Liquid Biopsy

Liquid biopsy acquired from bodily fluids near malignant cells provides valuable information without invasive tissue removal [25,26]. Especially for tumors that are difficult to approach, even a least invasive percutaneous biopsy may be uncomfortable and increase the risk of complications. Additionally, conventional biopsies may only cover a limited portion of the tumor, which may not accurately reflect it [27]. Blood is the most suitable bodily fluid for liquid biopsy, while urine, cerebrospinal fluid, and saliva may also be beneficial depending on the primary tumor [26]. Liquid biopsies from blood can be used for multiplexed cancer profiling analyses including circulating biomarkers (such as cross-linked type 1 collagen, bone sialoprotein, TRAcP5B, osteoprotegerin) and metabolites (such as pyridinoline and deoxypyridino). Three basic categories of actionable biological components may be acquired from a blood liquid biopsy, including CTCs, cell-free ctDNA, and EVs, or exosomes [28].

### 4.1. Circulating Tumor Cells

CTCs are tumor cells that enter the bloodstream. Ashworth initially recognized them almost 150 years ago, and they are now widely employed in therapeutic practice [29]. Isolating CTCs has been challenging due to their low number in the general circulation. However, new microfluidic platforms like the U.S. Food and Drug Administration (FDA)-approved Cell Search platform and next-generation sequencing allow for deep phenotyping of every isolated tumor cell [30]. Recent improvements enable liquid biopsies to reveal the genomic mosaicism, mutational landscape, epigenetics, and gene and protein expression of the original tumor.

### 4.2. Circulating Tumor DNA

The majority of ctDNA found in the general circulation is 180–200 bp in length, indicating that it originates from apoptotic and necrotic primary tumor cells [28]. Tumor DNA may reveal mutations and copy number variation, evaluating the need for specific-target treatment. For instance, the V600E mutation in BRAF is seen in several cancers such as metastatic colorectal cancer, melanoma, and papillary thyroid carcinoma [31,32,33,34]. Targeting the V600E form of BRAF with medicines such vemurafenib, dabrafenib, and trametinib may influence treatment choices [35].

### 4.3. Extracellular Vesicles (EVs)

EVs are lipid bilayer particles with a diameter of 30 to 1000 nm that may be divided into three types: apoptotic bodies, large EVs (microvesicles), and tiny EVs (exosomes). The biogenesis and biological function vary [36,37]. Cell-secreted EVs can include other biological components, such as DNA, RNA, and proteins, which can be used in determining a diagnosis and prognosis [36,38]. More EVs are produced in cancer cells than normal cells, and play an important role in bone metastases, osteosarcoma, and Ewing’s sarcoma [39,40]. In addition, normal and cancer cells show increased EV production due to hypoxia, increased intracellular calcium or pH, oxidative stress, ionizing radiation, and ultrasound [41]. Further, while RNA can degrade in circulation, encapsulating the RNA in EVs makes them more stable and amenable to transcriptional analyses [42]. Using various methods to gather biological data from patients’ blood may aid in accurate diagnosis, prognosis, and monitoring drug-resistant clones for informed treatment decisions [43]. Due to cost, scalability, repeatability, and separation procedures, bone liquid biopsy is not widely used in clinical trials, despite preclinical breakthroughs. However, it has shown substantial advances and preclinical and clinical uses in bone tumors including secondary and primary malignancies.

## 5. Liquid Biopsy in Bone Metastases

### 5.1. Circulating Tumor DNA

Circulating tumor DNA analysis may reveal the mutational landscape of metastatic disease and predict recurrence or response to therapy [44]. A retrospective study of primary breast cancer patients detected metastatic disease, including bone metastases, by measuring tumor-specific chromosomal rearrangements in ctDNA using droplet-based digital PCR technologies from plasma samples, nearly 1 year before clinical recurrence detection. The amount of ctDNA was directly proportional to disease progression. This suggests that ctDNA detection may be a useful technique for early metastasis detection. Liquid biopsy may also reveal minimum residual disease (MRD), indicating treatment and prognosis. Plasma tumor-associated ctDNA detection and analysis are effective indicators for identifying and monitoring MRD in breast cancer patients at high risk of recurrence [45,46]. Levels of ctDNA at baseline are linked to increased bone metastases and poor prognosis in non-small cell lung cancer (NSCLC) patients [47]. Late-stage NSCLC patients show increased ctDNA levels in patients with bone metastases [48], while higher ctDNA levels are detected in prostate cancer patients with visceral metastases than in those with bone metastases [49]. Detecting MRD using liquid biopsy is still in its infancy, and larger-scale longitudinal investigations are needed to investigate false positives and negative cases.

### 5.2. Circulating Tumor Cells

Circulating tumor cells have been shown to be beneficial for the diagnosis, prognosis, and monitoring of patients with metastatic breast cancer; in these patients, CTC detection and characterization has been linked to bone and hepatic metastases [50]. Multiple metastatic locations are associated with greater CTC counts, but bone-only metastatic breast cancer patients have lower CTC counts and improved prognoses [51,52]. Patients with one or two bone metastases show significantly fewer CTCs than those with more bone metastases [52].

CTCs exhibit a subset of metastasis-initiating cells expressing CD44, CD47, and c-MET. When transferred from a patient to immunocompromised animals, these cells cause metastases in the lungs, liver, and bones [53]. The discovery and quantification of CTCs may predict lung cancer prognosis, with high CTC counts as a predictor of bone metastases in advanced lung cancer patients [54,55] and in monitoring bone metastases in castration-resistant prostate cancer patients. Other studies indicate that CTC counts > 5 per 7.5 mL of blood predict bone metastases and worse overall survival [56,57,58]. Another study recommended a liquid biopsy method that detects and quantifies both CTCs and ctDNA simultaneously. Peripheral blood samples collected before and after treatment in a homogenous cohort of HER2-negative breast cancer patients [59] were examined prospectively in the COMET trial (NCT01745757). Compared to non-metastatic patients, greater CTCs and ctDNA mutations in tumor protein 53, phosphatidylinositol-4,5-bisphosphate 3-kinase catalytic subunit alpha, and estrogen receptor 1 (ESR1) genes were observed in bone, liver, and brain metastasis patients [59]. Similarly, results of other investigations have indicated that the CTCs-ctDNA signature is effective for the diagnosis and prognosis of metastatic breast cancer [60,61,62,63].

### 5.3. Extracellular Vesicles Cargo and miRNAs

Bhadresha et al. [64] identified 15 genes consistently upregulated in bone metastasis patients. Using serum-derived EVs, five genes (HSP90AA1, osteopontin, IL-3, VEGFA, and protein tyrosine kinase 2) were upregulated in breast and lung cancer patients with bone metastasis. The results indicated that EV-derived mRNA may be used to detect early bone metastases in cases of breast or lung cancer [64]. Plasma-derived EV miRNAs (hsa-miR-574-5p, 328-3p and 423-3p) from NSCLC patients were examined retrospectively as early biomarkers of bone metastases [65]. In that study, Yang et al. observed that EV-derived miR-181a-5p- is increased in prostate cancer patients with bone metastases. Bryant et al. obtained similar results for miR-141 and miR-375 [66]. Prostate microparticle-specific EVs have been reported to be more prevalent in metastatic prostate cancer than in no-metastatic disease and outperformed the FDA-approved CellSearch system in predicting CTCs [67]. ExoDX, a recently evaluated urine exosomal gene expression platform, outperformed the gold standard in predicting high-grade prostate cancer in patients with uncertain PSA scores (ROC AUC 0.7 vs. 0.62) and identifying benign prostate hyperplasia, reducing unnecessary biopsies [68].

## 6. Liquid Biopsy in Bone Sarcomas

### 6.1. Circulating Tumor DNA

Few studies have examined plasma-derived ctDNAs from osteosarcoma patients [69,70]. One study examined somatic mutations with tumor burden and prognosis using targeted next-generation sequencing (NGS) to detect tumor-specific somatic alterations in plasma samples at various stages of treatment, allowing for disease burden monitoring [69]. In the research of Shulman et al. [70], NGS hybrid capture assay ctDNA levels in peripheral blood samples of newly diagnosed localized osteosarcoma and Ewing sarcoma patients were linked to tumor burden, recurrence, and poor clinical outcomes; interestingly, ctDNA analysis revealed unexpected genetic characteristics of osteosarcoma, such as chromosomal arm 8q copy number increases [70]. Genetic mutations, including STAG2 and TP53 loss-of-function mutations, translocation events and fusion genes, have been identified in Ewing sarcoma patients, allowing for ctDNA monitoring of the bone malignancy [71]. In the study of Shulman et al. [70], ctDNA detection in plasma samples was linked to a poor clinical outcome in newly diagnosed Ewing sarcoma patients and revealed genomic information like EWSR1 fusion and STAG2 loss-of-function mutations [70]. Hayashi et al. observed that plasma EWSR1-FLI1 fusion gene levels are associated to tumor burden and therapeutic response in Ewing sarcoma patients, suggesting another potential use for liquid biopsy. In addition, EWS-FLI1 levels in the blood fall following chemotherapy or surgery and subsequently increase after tumor recurrence [72].

### 6.2. Circulating Tumor Cells

Osteosarcoma metastasis may be predicted by CTCs [73]. Li et al. [74] found additional CTCs in peripheral blood of metastatic osteosarcoma patients compared to those with localized disease in a prospective analysis. Additionally, CTC count has been shown to be negatively linked with the patient’s response following neoadjuvant chemotherapy [74]. Preclinical studies indicate that CTC count variations after treatment or surgery can indicate tumor sensitivity and metastasis [75,76]. An increased percentage of mesenchymal CTCs in peripheral blood of osteosarcoma patients after chemotherapy treatment has been linked to lower disease-free survival. This highlights the importance of monitoring changes in CTCs to assess treatment efficacy and detect disease recurrence or metastasis. In Ewing sarcoma patients, CTC characterization using tumor-specific markers (i.e., CD99 expression) and chromosomal translocations (e.g., EWSR1-FLI1 transcript fusion gene amplification) has been described [77,78]. In those patients, CTCs detected at diagnosis correlates with worse clinical outcomes and increased recurrence disease and metastasis [78,79].

### 6.3. Extracellular Vesicles Cargo and miRNAs

A liquid biopsy has been used to study EVs as diagnostic or prognostic serum indicators in osteosarcoma. RNA analysis of circulating EVs in metastatic osteosarcoma samples has revealed various transcriptome changes, offering a novel therapeutically useful method for tracking metastatic osteosarcoma [80]. Osteosarcoma patients’ peripheral blood contains miRNAs that are known to partly circulate within EVs and have oncogenic or antitumor suppressive functions. Several biomarkers, including miR-148a [81], miR-574-3p, miR-214, miR-335-5p, miR-491, miR-221, miR-191, and miR-421, are becoming important diagnostic and prognostic indicators, while osteosarcoma patients have lower levels of miR-124, miR-101, and miR-195 in their blood compared to those of healthy persons [82,83,84,85,86]. These data may be used to develop a prognostic approach for osteosarcoma employing a mix of miRNAs.

Recently, Ewing sarcoma has been studied for circulating miRNAs. One example of a circulating miRNA linked to Ewing sarcoma development is miR-125b, which has been shown to be lower in patients’ blood after surgery compared to that in healthy controls [87]. Decreased expression of this gene has been linked to a poor chemotherapy response in the same study [87]. Research is now focusing on Ewing sarcoma-derived EVs cargo as a predictive biomarker source, notably protein content. Ewing sarcoma-derived tiny EVs may be biomarked by CD99, HINT1, and NGFR membrane proteins according to Samuel et al. They used these EV surface proteins to immuno-enrich Ewing sarcoma-associated tiny EVs and identify EWS-FLI1 and EWS-ERG fusion transcripts in plasma from localized and metastatic patients [88].

## 7. Clinical Implication of Liquid Biopsy in Monitoring Drug Resistance

### 7.1. Liquid Biopsy in Chemoresistant Primary and Secondary Bone Tumors

Recent research indicates that the tumor secretome, including DNA fragments from drug-resistant cells with mutations, is abundant in plasma, making blood-based liquid biopsy crucial [89]. Recent studies on plasma samples of small patient cohorts have identified resistance mutations during treatment. While the data is clinically informative about the therapy response, it has not yet been fully validated in clinical practice. Quantification and analysis of ctDNA are effective tools for analyzing various tumor types [90]. The potential of ctDNA for monitoring treatment efficacy can be demonstrated in the finding that breast cancer patients with metastases, treated with aromatase inhibitors, and carrying ESR1 mutations in ctDNA are likely to show resistance to endocrine therapy and experience shorter progression-free survival [91].

Liquid biopsy can also identify biomarkers linked to CDK inhibitor resistance and predict metastatic disease in advanced breast cancer patients with hormone receptor-positive/HER2-. Patients receiving CDK inhibitor and endocrine therapy show specific ctDNA mutations, such as retinoblastoma, ESR1, fibroblast growth factor receptor 1, or phosphatidylinositol-4,5-bisphosphate 3-kinase catalytic subunit alpha alterations [92,93,94], potentially influencing disease outcomes and therapeutic decisions. Additionally, detecting and quantifying CTC acquired resistance may serve as a predictive marker for treatment outcomes [95]. In castration-resistant prostate cancer patients treated with docetaxel, CTC count in blood is a reliable indication of therapy sensitivity and survival [96].

Osteosarcoma treatment difficulty stems from genetic instability and the emergence of chemotherapy resistance after selection pressure; low levels of miR-375 in osteosarcoma patients have been associated with poor response to preoperative chemotherapy [97]. Recent research has linked tumor-associated miRNAs to osteosarcoma chemoresistance, including miR-491, which is decreased in serum from patients compared to that in healthy controls. This decrease is linked to increased metastasis, poor chemoresponse, and lower survival rates [98]. Serum miR-21 levels are considerably greater in osteosarcoma patients compared to those in controls and are associated with advanced Enneking stage and chemotherapy resistance [99]. Reduced miR-125b levels in Ewing sarcoma patients are linked to poor treatment response and chemoresistance development [87].

### 7.2. Implication of Extracellular Vesicles in Chemoresistance

EVs play a crucial role in drug resistance transmission [100], making them valuable for monitoring its emergence during therapy. miRNAs may represent indicators for chemoresistance. High quantities of membrane transporter pump P-glycoprotein in EVs from doxorubicin-resistant osteosarcoma MG63 cells facilitate horizontal transmission of resistance to susceptible cancer cells [101]. Additionally, miR-25-3p overexpression in osteosarcoma patients’ blood has been linked to tumor development and medication resistance [102]. The levels of miR-222-3p in EVs derived from NSCLC patients’ blood may predict sensitivity to gemcitabine and identify individuals with advanced and resistant illness [103]. In addition, platinum-resistant NSCLC patients have increased EV-derived miR-425-3p in their blood compared to that of platinum-sensitive individuals [104]. Research indicates that miR-222 from doxorubicin-resistant breast cancer cells is transported by EVs to M2 macrophages, inducing polarization. In contrast, miR-222 overexpression suppresses the expression of the tensin homolog gene and phosphatase activity, leading to Akt phosphorylation and activation, which promotes the proliferation of cancer cells, as well as their migration and invasion through positive feedback. EVs from the plasma of chemoresistant breast cancer patients show increased levels of miRNA-222 [105], while paclitaxel-treated cells from the human osteotropic breast cancer cell line MDA-MB-231 release EVs enriched in Survivin [106]. Unexpectedly, EVs can directly inhibit anti-neoplastic drugs; EVs from HER2-positive breast cancer patients behave as decoy receptors for trastuzumab, affecting its activity [107].

EVs generated by cancer cells include HER2 on their surface that is bound by trastuzumab systemically, reducing the quantity of antibodies available for cell binding. Yang et al. identified increased GSTP1 mRNA levels in EVs from non-responding breast cancer patients treated with neoadjuvant chemotherapy compared to those of responders. GSTP1-containing EVs have been shown to transmit drug resistance horizontally, suggesting their potential as negative predictors of chemoresistance and clinical outcomes in breast cancer patients receiving anthracycline/taxane treatment [108].

Transient receptor potential channel 5 mRNA in EVs isolated from the blood of metastatic breast cancer patients may predict chemoresistance [109]. Kharaziha et al. [110] identified MDR-1, MDR-3, endophilin-A2, and poly(A) binding protein 4 as enriched proteins in EVs from both prostate cancer cells resistant to docetaxel and castration-resistant prostate cancer patient serum, suggesting potential as biomarkers for therapeutic response or resistance [110]. The field is promising in cancer research, although larger longitudinal studies are necessary to confirm the effect of biomarkers.

### 7.3. Factors Hindering the Clinical Applications of Liquid Biopsies

Despite the potential of liquid biopsies, difficulties must be solved before broad clinical use. Due to the sensitivity of the approaches, even little variations in sample collection or processing may significantly impact the final output. The use of serum instead of plasma may increase cell-free DNA from other sources, lowering the diagnostic power of NGS-based tests, particularly for uncommon variations [111]. Lifestyle variables may impact cell-free DNA release in the bloodstream, creating a complex set of confounding factors that are challenging to detect and define [112]. CTCs are uncommon and difficult to acquire, and although the CellSearch technique offers a uniform approach, it is limited in its viability. They are only suitable for DNA and FACS/Immunofluorescence investigations, not RNA-based or functional experiments such as patient-derived xenografts or in vitro drug sensitivity testing [113,114]. CTC analysis also has limitations similar to those of conventional biopsies, since it may not reflect the complete tumor, but rather a subset of cells that survived in the circulation. An experiment is underway to partly address this problem by selecting various blood collection locations. Current research suggests that arterial blood and blood from near the main tumor may provide more CTCs [115,116].

Despite efforts to develop guidelines for sample treatment, liquid biopsy requires training, specialized facilities, and expertise in the interpretation of results [117]. EVs have unique preanalytical obstacles, in addition to the broader issues mentioned above. They represent a new source of biological information; however, further research is needed to develop EVs as a liquid biopsy. EV isolation is a prime example, as several methods have been investigated for isolating EVs [118]. Unfortunately, there is no perfect strategy for EV separation, and findings may vary based on the investigator’s approach [118]. Additionally, lifestyle variables may also promote EV release, making tumor-specific exosome identification difficult [41].

## 8. Conclusions

Traditional biopsy methods balance diagnostic accuracy with patient morbidity, tumor spread, and therapeutic interference. Percutaneous core-needle biopsies are preferred for their reduced contamination risk and cost. Imaging-guided musculoskeletal biopsies improve accuracy and minimize complications. The necessity of orthopedic oncologists, diagnostic and interventional radiologists, and pathologists working together is shown by the agreement that minor incisional biopsies should be performed when percutaneous biopsies fail. In parallel, liquid biopsies either as a single procedure or combined with standard biopsy procedures may revolutionize clinical oncology, although they still need to be refined before they become regular.

In addition to improving patient quality of life and life expectancy, liquid biopsies may save healthcare expenses. Recent cost–consequence assessments demonstrate that combining tissue and liquid biopsies may cut healthcare costs. In addition to diagnosing bone metastases, liquid biopsies may detect risk factors and enable preventative treatments. Although CTCs have FDA permission for prognostic use in certain metastatic malignancies, liquid biopsies may soon include ctDNA and tumor-derived EVs. These components provide real-time monitoring of tumor growth and chemoresistance by genetically reflecting the whole tumor, making identification and characterization simpler. EVs are also linked to cancer and chemoresistance and might be addressed. Liquid biopsies will expand because of next-generation sequencing and single-EV sequencing. To maximize these approaches’ benefits, oncologists, diagnostic and interventional radiologists, pathologists, and researchers must work together to enhance diagnosis, patient outcomes, and healthcare costs.

## Figures and Tables

**Table 1:** The principles of traditional biopsy techniques.

Principles	Comments
Biopsy should be the last step of staging	A lesion may be a primary bone sarcoma that may require a biopsy technique that allows for future limb salvage surgery. Another more accessible lesion may be found. If renal cell carcinoma likely, consider preoperative embolization. If the diagnosis of multiple myeloma is made by laboratory studies, an unnecessary biopsy will be avoided. The pathological diagnosis will be more accurate if aided by appropriate imaging studies. The pathologist and surgeon may be more assured of a diagnosis of metastasis made on frozen section analysis if supported by the preoperative evaluation.
Biopsy without delay	Perform early biopsy with appropriate oncological principles only after clinical, laboratory, and imaging examinations are complete.
Biopsy at a reference tumor center	The tumor patient should be referred for biopsy to the institution where definitive treatment will be performed.
Biopsy principles are independent of the biopsy technique	The principles of biopsy are the same regardless of the biopsy technique.
Biopsy contraindications	Uncorrected coagulopathy. Inability to obtain a safe access route. Inability to obtain patient consent. Hydatid disease.
Open incisional biopsy	When adequate tissue sample cannot be obtained with closed biopsy. When accurate histological diagnosis and grading is required to decide for preoperative chemotherapy or radiation therapy. When closed biopsy does not correlate with the clinical presentation and imaging. If non-diagnostic closed biopsy. Can be used with frozen sections. Use the smallest incision that is compatible with obtaining adequate specimen. Careful hemostasis (use tourniquet; very rarely use drains).
Open excisional biopsy	Any soft tissue neoplasm highly likely is a sarcoma if it is (a) deep to the fascia, and (b) >3–5 cm in its greatest dimension. Perform only if the lesion can be excised with wide margins, otherwise, any such lesion should be biopsied.
Open biopsy approach	Longitudinal incision. Contamination of one anatomic compartment. Excisable biopsy tract. Meticulous hemostasis. Drain, if necessary, exit in line with skin incision.
Closed biopsy	The gold standard, especially if guided. Minimally invasive, high accuracy (no difference with open biopsy), cost effective, rare complications (<11%).
Fine needle biopsy	No role in musculoskeletal tumors; high rate of false negative results, cytology samples are not always adequate for cytogenetic, molecular or immunohisto studies, recommended for documentation of metastases and local or distant tumor recurrence where the cytology findings can be compared with prior histology.
Discuss with the radiologist and pathologist	The surgeon, the radiologist, and the pathologist must examine the imaging studies. The pathologist must ask for further tissue analysis if pathology report gives non-specific tissue description. Repeat the biopsy if the histological diagnosis is in doubt or inconsistent with the suspected clinical and/or imaging diagnosis.

## References

[1] Kasraeian S., Allison D.C., Ahlmann E.R., Fedenko A.N., Menendez L.R. (2010). A Comparison of Fine-Needle Aspiration, Core Biopsy, and Surgical Biopsy in the Diagnosis of Extremity Soft Tissue Masses. Clin. Orthop. Relat. Res..

[2] Bickels J., Jelinek J.S., Shmookler B.M., Neff R.S., Malawer M.M. (1999). Biopsy of Musculoskeletal Tumors. Current Concepts. Clin. Orthop. Relat. Res..

[3] Mankin H.J., Gebhardt M.C., Jennings L.C., Springfield D.S., Tomford W.W. (1996). Long-Term Results of Allograft Replacement in the Management of Bone Tumors. Clin. Orthop. Relat. Res..

[4] Le H.B.Q., Lee S.T., Munk P.L. (2010). Image-Guided Musculoskeletal Biopsies. Semin. Intervent. Radiol..

[5] Pohlig F., Kirchhoff C., Lenze U., Schauwecker J., Burgkart R., Rechl H., von Eisenhart-Rothe R. (2012). Percutaneous Core Needle Biopsy versus Open Biopsy in Diagnostics of Bone and Soft Tissue Sarcoma: A Retrospective Study. Eur. J. Med. Res..

[6] Adams S.C., Potter B.K., Pitcher D.J., Temple H.T. (2010). Office-Based Core Needle Biopsy of Bone and Soft Tissue Malignancies: An Accurate Alternative to Open Biopsy with Infrequent Complications. Clin. Orthop. Relat. Res..

[7] Yang Y.J., Damron T.A. (2004). Comparison of Needle Core Biopsy and Fine-Needle Aspiration for Diagnostic Accuracy in Musculoskeletal Lesions. Arch. Pathol. Lab. Med..

[8] Serpell J.W., Pitcher M.E. (1998). Pre-Operative Core Biopsy of Soft-Tissue Tumours Facilitates Their Surgical Management. Aust. N. Z. J. Surg..

[9] López J.I., Del Cura J.L., Zabala R., Bilbao F.J. (2005). Usefulness and Limitations of Ultrasound-Guided Core Biopsy in the Diagnosis of Musculoskeletal Tumours. APMIS.

[10] Skrzynski M.C., Biermann J.S., Montag A., Simon M.A. (1996). Diagnostic Accuracy and Charge-Savings of Outpatient Core Needle Biopsy Compared with Open Biopsy of Musculoskeletal Tumors. J. Bone Joint Surg. Am..

[11] Rimondi E., Staals E.L., Errani C., Bianchi G., Casadei R., Alberghini M., Malaguti M.C., Rossi G., Durante S., Mercuri M. (2008). Percutaneous CT-Guided Biopsy of the Spine: Results of 430 Biopsies. Eur. Spine J..

[12] Liu J.-C., Chiou H.-J., Chen W.-M., Chou Y.-H., Chen T.-H., Chen W., Yen C.-C., Chiu S.-Y., Chang C.-Y. (2004). Sonographically Guided Core Needle Biopsy of Soft Tissue Neoplasms. J. Clin. Ultrasound.

[13] Serpell J.W., Fish S.H., Fisher C., Thomas J.M. (1992). The Diagnosis of Soft Tissue Tumours. Ann. R. Coll. Surg. Engl..

[14] Rydholm A., Alvegård T., Berg N.O., Dawiskiba Z., Egund N., Idvall I., Pettersson H., Rööser B., Willén H., Akerman M. (1988). Preoperative Diagnosis of Soft Tissue Tumours. Int. Orthop..

[15] Heslin M.J., Lewis J.J., Woodruff J.M., Brennan M.F. (1997). Core Needle Biopsy for Diagnosis of Extremity Soft Tissue Sarcoma. Ann. Surg. Oncol..

[16] Clark C.R., Morgan C., Sonstegard D.A., Matthews L.S. (1977). The Effect of Biopsy-Hole Shape and Size on Bone Strength. J. Bone Joint Surg Am..

[17] Sung K.-S., Seo S.-W., Shon M.-S. (2009). The Diagnostic Value of Needle Biopsy for Musculoskeletal Lesions. Int. Orthop..

[18] Rajeswaran G., Malik Q., Saifuddin A. (2013). The Role of Needle Biopsy for Focal Bone Lesions with Complete Fluid-Fluid Levels on Magnetic Resonance Imaging. Skelet. Radiol..

[19] Lehotska V. (2005). Soft-Tissue Tumors-Role of Diagnostic Imaging. Bratisl. Lek. Listy..

[20] Rougraff B.T., Aboulafia A., Biermann J.S., Healey J. (2009). Biopsy of Soft Tissue Masses: Evidence-Based Medicine for the Musculoskeletal Tumor Society. Clin. Orthop. Relat. Res..

[21] Iwamoto Y. (1999). Diagnosis and Treatment of Soft Tissue Tumors. J. Orthop. Sci..

[22] Mankin H.J., Hornicek F.J., DeLaney T.F., Harmon D.C., Schiller A.L. (2012). Pleomorphic Spindle Cell Sarcoma (PSCS) Formerly Known as Malignant Fibrous Histiocytoma (MFH): A Complex Malignant Soft-Tissue Tumor. Musculoskelet. Surg..

[23] Al-Nammari S.S., Danesh A., Mussa M., Al-Hadithy N. (2013). The Portrayal of Bone Tumours in the Press. Musculoskelet. Surg..

[24] Ng V.Y., Thomas K., Crist M., Wakely P.E., Mayerson J. (2010). Fine Needle Aspiration for Clinical Triage of Extremity Soft Tissue Masses. Clin. Orthop. Relat. Res..

[25] Eslami-S Z., Cortés-Hernández L.E., Alix-Panabières C. (2020). The Metastatic Cascade as the Basis for Liquid Biopsy Development. Front. Oncol..

[26] Palmirotta R., Lovero D., Cafforio P., Felici C., Mannavola F., Pellè E., Quaresmini D., Tucci M., Silvestris F. (2018). Liquid Biopsy of Cancer: A Multimodal Diagnostic Tool in Clinical Oncology. Ther. Adv. Med. Oncol..

[27] Li X., Seebacher N.A., Hornicek F.J., Xiao T., Duan Z. (2018). Application of Liquid Biopsy in Bone and Soft Tissue Sarcomas: Present and Future. Cancer Lett..

[28] Perakis S., Speicher M.R. (2017). Emerging Concepts in Liquid Biopsies. BMC Med..

[29] de Wit S., van Dalum G., Terstappen L.W.M.M. (2014). Detection of Circulating Tumor Cells. Scientifica.

[30] Yang C., Xia B.-R., Jin W.-L., Lou G. (2019). Circulating Tumor Cells in Precision Oncology: Clinical Applications in Liquid Biopsy and 3D Organoid Model. Cancer Cell Int..

[31] Mauri G., Bonazzina E., Amatu A., Tosi F., Bencardino K., Gori V., Massihnia D., Cipani T., Spina F., Ghezzi S. (2021). The Evolutionary Landscape of Treatment for BRAFV600E Mutant Metastatic Colorectal Cancer. Cancers.

[32] Ascierto P.A., Kirkwood J.M., Grob J.-J., Simeone E., Grimaldi A.M., Maio M., Palmieri G., Testori A., Marincola F.M., Mozzillo N. (2012). The Role of BRAF V600 Mutation in Melanoma. J. Transl. Med..

[33] Pellegrini C., Di Nardo L., Cipolloni G., Martorelli C., De Padova M., Antonini A., Maturo M.G., Del Regno L., Strafella S., Micantonio T. (2018). Heterogeneity of BRAF, NRAS, and TERT Promoter Mutational Status in Multiple Melanomas and Association with MC1R Genotype: Findings from Molecular and Immunohistochemical Analysis. J. Mol. Diagn..

[34] Rashid F.A., Bhat G.H., Khan M.S., Tabassum S., Bhat M.H. (2022). Variations in MAP Kinase Gladiators and Risk of Differentiated Thyroid Carcinoma. Mol. Clin. Oncol..

[35] Crowley E., Di Nicolantonio F., Loupakis F., Bardelli A. (2013). Liquid Biopsy: Monitoring Cancer-Genetics in the Blood. Nat. Rev. Clin. Oncol..

[36] Colombo M., Raposo G., Théry C. (2014). Biogenesis, Secretion, and Intercellular Interactions of Exosomes and Other Extracellular Vesicles. Annu. Rev. Cell Dev. Biol..

[37] Doyle L.M., Wang M.Z. (2019). Overview of Extracellular Vesicles, Their Origin, Composition, Purpose, and Methods for Exosome Isolation and Analysis. Cells.

[38] Stevic I., Buescher G., Ricklefs F.L. (2020). Monitoring Therapy Efficiency in Cancer through Extracellular Vesicles. Cells.

[39] Green T.M., Alpaugh M.L., Barsky S.H., Rappa G., Lorico A. (2015). Breast Cancer-Derived Extracellular Vesicles: Characterization and Contribution to the Metastatic Phenotype. Biomed. Res. Int..

[40] Taverna S., Giusti I., D’Ascenzo S., Pizzorno L., Dolo V. (2020). Breast Cancer Derived Extracellular Vesicles in Bone Metastasis Induction and Their Clinical Implications as Biomarkers. Int. J. Mol. Sci..

[41] Ludwig N., Whiteside T.L., Reichert T.E. (2019). Challenges in Exosome Isolation and Analysis in Health and Disease. Int. J. Mol. Sci..

[42] Kim K.M., Abdelmohsen K., Mustapic M., Kapogiannis D., Gorospe M. (2017). RNA in Extracellular Vesicles. Wiley Interdiscip. Rev. RNA.

[43] Siravegna G., Marsoni S., Siena S., Bardelli A. (2017). Integrating Liquid Biopsies into the Management of Cancer. Nat. Rev. Clin. Oncol..

[44] Incorvaia L., Castiglia M., Perez A., Massihnia D., Caruso S., Altintas S., Calò V., Russo A., Russo A., Giordano A., Rolfo C. (2017). Liquid Biopsy in Breast Cancer. Liquid Biopsy in Cancer Patients: The Hand Lens for Tumor Evolution.

[45] Garcia-Murillas I., Schiavon G., Weigelt B., Ng C., Hrebien S., Cutts R.J., Cheang M., Osin P., Nerurkar A., Kozarewa I. (2015). Mutation Tracking in Circulating Tumor DNA Predicts Relapse in Early Breast Cancer. Sci. Transl. Med..

[46] Beaver J.A., Jelovac D., Balukrishna S., Cochran R., Croessmann S., Zabransky D.J., Wong H.Y., Toro P.V., Cidado J., Blair B.G. (2014). Detection of Cancer DNA in Plasma of Patients with Early-Stage Breast Cancer. Clin. Cancer Res..

[47] Pécuchet N., Zonta E., Didelot A., Combe P., Thibault C., Gibault L., Lours C., Rozenholc Y., Taly V., Laurent-Puig P. (2016). Base-Position Error Rate Analysis of Next-Generation Sequencing Applied to Circulating Tumor DNA in Non-Small Cell Lung Cancer: A Prospective Study. PLoS Med..

[48] Jia J., Huang B., Zhuang Z., Chen S. (2018). Circulating Tumor DNA as Prognostic Markers for Late Stage NSCLC with Bone Metastasis. Int. J. Biol. Markers.

[49] Vandekerkhove G., Struss W.J., Annala M., Kallio H.M.L., Khalaf D., Warner E.W., Herberts C., Ritch E., Beja K., Loktionova Y. (2019). Circulating Tumor DNA Abundance and Potential Utility in De Novo Metastatic Prostate Cancer. Eur. Urol..

[50] Bidard F.-C., Peeters D.J., Fehm T., Nolé F., Gisbert-Criado R., Mavroudis D., Grisanti S., Generali D., Garcia-Saenz J.A., Stebbing J. (2014). Clinical Validity of Circulating Tumour Cells in Patients with Metastatic Breast Cancer: A Pooled Analysis of Individual Patient Data. Lancet Oncol..

[51] Moussavi-Harami S.F., Wisinski K.B., Beebe D.J. (2014). Circulating Tumor Cells in Metastatic Breast Cancer: A Prognostic and Predictive Marker. J. Patient Cent. Res. Rev..

[52] De Giorgi U., Valero V., Rohren E., Mego M., Doyle G.V., Miller M.C., Ueno N.T., Handy B.C., Reuben J.M., Macapinlac H.A. (2010). Circulating Tumor Cells and Bone Metastases as Detected by FDG-PET/CT in Patients with Metastatic Breast Cancer. Ann. Oncol..

[53] Baccelli I., Schneeweiss A., Riethdorf S., Stenzinger A., Schillert A., Vogel V., Klein C., Saini M., Bäuerle T., Wallwiener M. (2013). Identification of a Population of Blood Circulating Tumor Cells from Breast Cancer Patients That Initiates Metastasis in a Xenograft Assay. Nat. Biotechnol..

[54] Cheng M., Liu L., Yang H.-S., Liu G.-F. (2014). Circulating Tumor Cells Are Associated with Bone Metastasis of Lung Cancer. Asian Pac. J. Cancer Prev..

[55] Krebs M.G., Sloane R., Priest L., Lancashire L., Hou J.-M., Greystoke A., Ward T.H., Ferraldeschi R., Hughes A., Clack G. (2011). Evaluation and Prognostic Significance of Circulating Tumor Cells in Patients with Non-Small-Cell Lung Cancer. J. Clin. Oncol..

[56] Shaffer D.R., Leversha M.A., Danila D.C., Lin O., Gonzalez-Espinoza R., Gu B., Anand A., Smith K., Maslak P., Doyle G.V. (2007). Circulating Tumor Cell Analysis in Patients with Progressive Castration-Resistant Prostate Cancer. Clin. Cancer Res..

[57] de Bono J.S., Scher H.I., Montgomery R.B., Parker C., Miller M.C., Tissing H., Doyle G.V., Terstappen L.W.W.M., Pienta K.J., Raghavan D. (2008). Circulating Tumor Cells Predict Survival Benefit from Treatment in Metastatic Castration-Resistant Prostate Cancer. Clin. Cancer Res..

[58] Helo P., Cronin A.M., Danila D.C., Wenske S., Gonzalez-Espinoza R., Anand A., Koscuiszka M., Väänänen R.-M., Pettersson K., Chun F.K.-H. (2009). Circulating Prostate Tumor Cells Detected by Reverse Transcription-PCR in Men with Localized or Castration-Refractory Prostate Cancer: Concordance with CellSearch Assay and Association with Bone Metastases and with Survival. Clin. Chem..

[59] Bortolini Silveira A., Bidard F.-C., Tanguy M.-L., Girard E., Trédan O., Dubot C., Jacot W., Goncalves A., Debled M., Levy C. (2021). Multimodal Liquid Biopsy for Early Monitoring and Outcome Prediction of Chemotherapy in Metastatic Breast Cancer. NPJ Breast Cancer.

[60] Kodahl A.R., Ehmsen S., Pallisgaard N., Jylling A.M.B., Jensen J.D., Laenkholm A.-V., Knoop A.S., Ditzel H.J. (2018). Correlation between Circulating Cell-Free PIK3CA Tumor DNA Levels and Treatment Response in Patients with PIK3CA-Mutated Metastatic Breast Cancer. Mol. Oncol..

[61] Dawson S.-J., Tsui D.W.Y., Murtaza M., Biggs H., Rueda O.M., Chin S.-F., Dunning M.J., Gale D., Forshew T., Mahler-Araujo B. (2013). Analysis of Circulating Tumor DNA to Monitor Metastatic Breast Cancer. N. Engl. J. Med..

[62] Wang P., Bahreini A., Gyanchandani R., Lucas P.C., Hartmaier R.J., Watters R.J., Jonnalagadda A.R., Trejo Bittar H.E., Berg A., Hamilton R.L. (2016). Sensitive Detection of Mono-and Polyclonal ESR1 Mutations in Primary Tumors, Metastatic Lesions, and Cell-Free DNA of Breast Cancer Patients. Clin. Cancer Res..

[63] Rossi G., Mu Z., Rademaker A.W., Austin L.K., Strickland K.S., Costa R.L.B., Nagy R.J., Zagonel V., Taxter T.J., Behdad A. (2018). Cell-Free DNA and Circulating Tumor Cells: Comprehensive Liquid Biopsy Analysis in Advanced Breast Cancer. Clin. Cancer Res..

[64] Bhadresha K.P., Patel M., Jain N.K., Rawal R.M. (2021). A Predictive Biomarker Panel for Bone Metastases: Liquid Biopsy Approach. J. Bone Oncol..

[65] Yang X.-R., Pi C., Yu R., Fan X.-J., Peng X.-X., Zhang X.-C., Chen Z.-H., Wu X., Shao Y., Wu Y.-L. (2021). Correlation of Exosomal microRNA Clusters with Bone Metastasis in Non-Small Cell Lung Cancer. Clin. Exp. Metastasis.

[66] Bryant R.J., Pawlowski T., Catto J.W.F., Marsden G., Vessella R.L., Rhees B., Kuslich C., Visakorpi T., Hamdy F.C. (2012). Changes in Circulating microRNA Levels Associated with Prostate Cancer. Br. J. Cancer.

[67] Biggs C.N., Siddiqui K.M., Al-Zahrani A.A., Pardhan S., Brett S.I., Guo Q.Q., Yang J., Wolf P., Power N.E., Durfee P.N. (2016). Prostate Extracellular Vesicles in Patient Plasma as a Liquid Biopsy Platform for Prostate Cancer Using Nanoscale Flow Cytometry. Oncotarget.

[68] McKiernan J., Donovan M.J., Margolis E., Partin A., Carter B., Brown G., Torkler P., Noerholm M., Skog J., Shore N. (2018). A Prospective Adaptive Utility Trial to Validate Performance of a Novel Urine Exosome Gene Expression Assay to Predict High-Grade Prostate Cancer in Patients with Prostate-Specific Antigen 2-10ng/Ml at Initial Biopsy. Eur. Urol..

[69] Barris D.M., Weiner S.B., Dubin R.A., Fremed M., Zhang X., Piperdi S., Zhang W., Maqbool S., Gill J., Roth M. (2018). Detection of Circulating Tumor DNA in Patients with Osteosarcoma. Oncotarget.

[70] Shulman D.S., Klega K., Imamovic-Tuco A., Clapp A., Nag A., Thorner A.R., Van Allen E., Ha G., Lessnick S.L., Gorlick R. (2018). Detection of Circulating Tumour DNA Is Associated with Inferior Outcomes in Ewing Sarcoma and Osteosarcoma: A Report from the Children’s Oncology Group. Br. J. Cancer.

[71] Shukla N.N., Patel J.A., Magnan H., Zehir A., You D., Tang J., Meng F., Samoila A., Slotkin E.K., Ambati S.R. (2017). Plasma DNA-Based Molecular Diagnosis, Prognostication, and Monitoring of Patients with *EWSR1* Fusion-Positive Sarcomas. JCO Precis. Oncol..

[72] Hayashi M., Chu D., Meyer C.F., Llosa N.J., McCarty G., Morris C.D., Levin A.S., Wolinsky J.-P., Albert C.M., Steppan D.A. (2016). Highly Personalized Detection of Minimal Ewing Sarcoma Disease Burden from Plasma Tumor DNA. Cancer.

[73] Zhang H., Gao P., Xiao X., Heger M., Geng L., Fan B., Yuan Y., Huang C., Chen G., Liu Y. (2017). A Liquid Biopsy-Based Method for the Detection and Quantification of Circulating Tumor Cells in Surgical Osteosarcoma Patients. Int. J. Oncol..

[74] Li M., Lu Y., Long Z., Li M., Kong J., Chen G., Wang Z. (2019). Prognostic and Clinicopathological Significance of Circulating Tumor Cells in Osteosarcoma. J. Bone Oncol..

[75] Wu Z.-J., Tan J.-C., Qin X., Liu B., Yuan Z.-C. (2018). Significance of Circulating Tumor Cells in Osteosarcoma Patients Treated by Neoadjuvant Chemotherapy and Surgery. Cancer Manag. Res..

[76] Chalopin A., Tellez-Gabriel M., Brown H.K., Vallette F., Heymann M.-F., Gouin F., Heymann D. (2018). Isolation of Circulating Tumor Cells in a Preclinical Model of Osteosarcoma: Effect of Chemotherapy. J. Bone Oncol..

[77] Benini S., Gamberi G., Cocchi S., Garbetta J., Alberti L., Righi A., Gambarotti M., Picci P., Ferrari S. (2018). Detection of Circulating Tumor Cells in Liquid Biopsy from Ewing Sarcoma Patients. Cancer Manag. Res..

[78] Schleiermacher G., Peter M., Oberlin O., Philip T., Rubie H., Mechinaud F., Sommelet-Olive D., Landman-Parker J., Bours D., Michon J. (2003). Increased Risk of Systemic Relapses Associated with Bone Marrow Micrometastasis and Circulating Tumor Cells in Localized Ewing Tumor. J. Clin. Oncol..

[79] Hayashi M., Zhu P., McCarty G., Meyer C.F., Pratilas C.A., Levin A., Morris C.D., Albert C.M., Jackson K.W., Tang C.-M. (2017). Size-Based Detection of Sarcoma Circulating Tumor Cells and Cell Clusters. Oncotarget.

[80] Bao Q., Gong L., Wang J., Wen J., Shen Y., Zhang W. (2018). Extracellular Vesicle RNA Sequencing Reveals Dramatic Transcriptomic Alterations Between Metastatic and Primary Osteosarcoma in a Liquid Biopsy Approach. Ann. Surg. Oncol..

[81] Ma W., Zhang X., Chai J., Chen P., Ren P., Gong M. (2014). Circulating miR-148a Is a Significant Diagnostic and Prognostic Biomarker for Patients with Osteosarcoma. Tumor Biol..

[82] Wang T., Ji F., Dai Z., Xie Y., Yuan D. (2015). Increased Expression of microRNA-191 as a Potential Serum Biomarker for Diagnosis and Prognosis in Human Osteosarcoma. Cancer Biomark..

[83] Zhou S., Wang B., Hu J., Zhou Y., Jiang M., Wu M., Qin L., Yang X. (2016). miR-421 Is a Diagnostic and Prognostic Marker in Patients with Osteosarcoma. Tumor Biol..

[84] Cong C., Wang W., Tian J., Gao T., Zheng W., Zhou C. (2018). Identification of Serum miR-124 as a Biomarker for Diagnosis and Prognosis in Osteosarcoma. Cancer Biomark..

[85] Yao Z.-S., Li C., Liang D., Jiang X.-B., Tang J.-J., Ye L.-Q., Yuan K., Ren H., Yang Z.-D., Jin D.-X. (2018). Diagnostic and Prognostic Implications of Serum miR-101 in Osteosarcoma. Cancer Biomark..

[86] Cai H., Zhao H., Tang J., Wu H. (2015). Serum miR-195 Is a Diagnostic and Prognostic Marker for Osteosarcoma. J. Surg. Res..

[87] Nie C.L., Ren W.H., Ma Y., Xi J.S., Han B. (2015). Circulating miR-125b as a Biomarker of Ewing’s Sarcoma in Chinese Children. Genet. Mol. Res..

[88] Samuel G., Crow J., Klein J.B., Merchant M.L., Nissen E., Koestler D.C., Laurence K., Liang X., Neville K., Staggs V. (2020). Ewing Sarcoma Family of Tumors-Derived Small Extracellular Vesicle Proteomics Identify Potential Clinical Biomarkers. Oncotarget.

[89] Russano M., Napolitano A., Ribelli G., Iuliani M., Simonetti S., Citarella F., Pantano F., Dell’Aquila E., Anesi C., Silvestris N. (2020). Liquid Biopsy and Tumor Heterogeneity in Metastatic Solid Tumors: The Potentiality of Blood Samples. J. Exp. Clin. Cancer Res..

[90] Bettegowda C., Sausen M., Leary R.J., Kinde I., Wang Y., Agrawal N., Bartlett B.R., Wang H., Luber B., Alani R.M. (2014). Detection of Circulating Tumor DNA in Early- and Late-Stage Human Malignancies. Sci. Transl. Med..

[91] Schiavon G., Hrebien S., Garcia-Murillas I., Cutts R.J., Pearson A., Tarazona N., Fenwick K., Kozarewa I., Lopez-Knowles E., Ribas R. (2015). Analysis of ESR1 Mutation in Circulating Tumor DNA Demonstrates Evolution during Therapy for Metastatic Breast Cancer. Sci. Transl. Med..

[92] O’Leary B., Hrebien S., Morden J.P., Beaney M., Fribbens C., Huang X., Liu Y., Bartlett C.H., Koehler M., Cristofanilli M. (2018). Early Circulating Tumor DNA Dynamics and Clonal Selection with Palbociclib and Fulvestrant for Breast Cancer. Nat. Commun..

[93] O’Leary B., Cutts R.J., Liu Y., Hrebien S., Huang X., Fenwick K., André F., Loibl S., Loi S., Garcia-Murillas I. (2018). The Genetic Landscape and Clonal Evolution of Breast Cancer Resistance to Palbociclib plus Fulvestrant in the PALOMA-3 Trial. Cancer Discov..

[94] Fribbens C., O’Leary B., Kilburn L., Hrebien S., Garcia-Murillas I., Beaney M., Cristofanilli M., Andre F., Loi S., Loibl S. (2016). Plasma ESR1 Mutations and the Treatment of Estrogen Receptor-Positive Advanced Breast Cancer. J. Clin. Oncol..

[95] Galardi F., De Luca F., Biagioni C., Migliaccio I., Curigliano G., Minisini A.M., Bonechi M., Moretti E., Risi E., McCartney A. (2021). Circulating Tumor Cells and Palbociclib Treatment in Patients with ER-Positive, HER2-Negative Advanced Breast Cancer: Results from a Translational Sub-Study of the TREnd Trial. Breast Cancer Res..

[96] Okegawa T., Itaya N., Hara H., Tambo M., Nutahara K. (2014). Circulating Tumor Cells as a Biomarker Predictive of Sensitivity to Docetaxel Chemotherapy in Patients with Castration-Resistant Prostate Cancer. Anticancer Res..

[97] Liu W., Zhao X., Zhang Y.-J., Fang G.-W., Xue Y. (2018). MicroRNA-375 as a Potential Serum Biomarker for the Diagnosis, Prognosis, and Chemosensitivity Prediction of Osteosarcoma. J. Int. Med. Res..

[98] Wang S.-N., Luo S., Liu C., Piao Z., Gou W., Wang Y., Guan W., Li Q., Zou H., Yang Z.-Z. (2017). miR-491 Inhibits Osteosarcoma Lung Metastasis and Chemoresistance by Targeting αB-Crystallin. Mol. Ther..

[99] Yuan J., Chen L., Chen X., Sun W., Zhou X. (2012). Identification of Serum microRNA-21 as a Biomarker for Chemosensitivity and Prognosis in Human Osteosarcoma. J. Int. Med. Res..

[100] Xavier C.P.R., Caires H.R., Barbosa M.A.G., Bergantim R., Guimarães J.E., Vasconcelos M.H. (2020). The Role of Extracellular Vesicles in the Hallmarks of Cancer and Drug Resistance. Cells.

[101] Torreggiani E., Roncuzzi L., Perut F., Zini N., Baldini N. (2016). Multimodal Transfer of MDR by Exosomes in Human Osteosarcoma. Int. J. Oncol..

[102] Yoshida A., Fujiwara T., Uotani K., Morita T., Kiyono M., Yokoo S., Hasei J., Nakata E., Kunisada T., Ozaki T. (2018). Clinical and Functional Significance of Intracellular and Extracellular microRNA-25-3p in Osteosarcoma. Acta Med. Okayama.

[103] Wei F., Ma C., Zhou T., Dong X., Luo Q., Geng L., Ding L., Zhang Y., Zhang L., Li N. (2017). Exosomes Derived from Gemcitabine-Resistant Cells Transfer Malignant Phenotypic Traits via Delivery of miRNA-222-3p. Mol. Cancer.

[104] Yuwen D., Ma Y., Wang D., Gao J., Li X., Xue W., Fan M., Xu Q., Shen Y., Shu Y. (2019). Prognostic Role of Circulating Exosomal miR-425-3p for the Response of NSCLC to Platinum-Based Chemotherapy. Cancer Epidemiol. Biomarkers Prev..

[105] Chen W.-X., Wang D.-D., Zhu B., Zhu Y.-Z., Zheng L., Feng Z.-Q., Qin X.-H. (2021). Exosomal miR-222 from Adriamycin-Resistant MCF-7 Breast Cancer Cells Promote Macrophages M2 Polarization via PTEN/Akt to Induce Tumor Progression. Aging.

[106] Kreger B.T., Johansen E.R., Cerione R.A., Antonyak M.A. (2016). The Enrichment of Survivin in Exosomes from Breast Cancer Cells Treated with Paclitaxel Promotes Cell Survival and Chemoresistance. Cancers.

[107] Ciravolo V., Huber V., Ghedini G.C., Venturelli E., Bianchi F., Campiglio M., Morelli D., Villa A., Della Mina P., Menard S. (2012). Potential Role of HER2-Overexpressing Exosomes in Countering Trastuzumab-Based Therapy. J. Cell Physiol..

[108] Yang S.-J., Wang D.-D., Li J., Xu H.-Z., Shen H.-Y., Chen X., Zhou S.-Y., Zhong S.-L., Zhao J.-H., Tang J.-H. (2017). Predictive Role of GSTP1-Containing Exosomes in Chemotherapy-Resistant Breast Cancer. Gene.

[109] Wang T., Ning K., Lu T.-X., Sun X., Jin L., Qi X., Jin J., Hua D. (2017). Increasing Circulating Exosomes-Carrying TRPC5 Predicts Chemoresistance in Metastatic Breast Cancer Patients. Cancer Sci..

[110] Kharaziha P., Chioureas D., Rutishauser D., Baltatzis G., Lennartsson L., Fonseca P., Azimi A., Hultenby K., Zubarev R., Ullén A. (2015). Molecular Profiling of Prostate Cancer Derived Exosomes May Reveal a Predictive Signature for Response to Docetaxel. Oncotarget.

[111] Pittella-Silva F., Chin Y.M., Chan H.T., Nagayama S., Miyauchi E., Low S.-K., Nakamura Y. (2020). Plasma or Serum: Which Is Preferable for Mutation Detection in Liquid Biopsy?. Clin. Chem..

[112] Merker J.D., Oxnard G.R., Compton C., Diehn M., Hurley P., Lazar A.J., Lindeman N., Lockwood C.M., Rai A.J., Schilsky R.L. (2018). Circulating Tumor DNA Analysis in Patients with Cancer: American Society of Clinical Oncology and College of American Pathologists Joint Review. J. Clin. Oncol..

[113] Wong K.H.K., Tessier S.N., Miyamoto D.T., Miller K.L., Bookstaver L.D., Carey T.R., Stannard C.J., Thapar V., Tai E.C., Vo K.D. (2017). Whole Blood Stabilization for the Microfluidic Isolation and Molecular Characterization of Circulating Tumor Cells. Nat. Commun..

[114] Fehm T.N., Meier-Stiegen F., Driemel C., Jäger B., Reinhardt F., Naskou J., Franken A., Neubauer H., Neves R.P.L., van Dalum G. (2018). Diagnostic Leukapheresis for CTC Analysis in Breast Cancer Patients: CTC Frequency, Clinical Experiences and Recommendations for Standardized Reporting. Cytom. Part A.

[115] Terai M., Mu Z., Eschelman D.J., Gonsalves C.F., Kageyama K., Chervoneva I., Orloff M., Weight R., Mastrangelo M.J., Cristofanilli M. (2015). Arterial Blood, Rather Than Venous Blood, is a Better Source for Circulating Melanoma Cells. EBioMedicine.

[116] Buscail E., Chiche L., Laurent C., Vendrely V., Denost Q., Denis J., Thumerel M., Lacorte J.-M., Bedel A., Moreau-Gaudry F. (2019). Tumor-Proximal Liquid Biopsy to Improve Diagnostic and Prognostic Performances of Circulating Tumor Cells. Mol. Oncol..

[117] Heidrich I., Ačkar L., Mossahebi Mohammadi P., Pantel K. (2021). Liquid Biopsies: Potential and Challenges. Int. J. Cancer.

[118] Brennan K., Martin K., FitzGerald S.P., O’Sullivan J., Wu Y., Blanco A., Richardson C., Mc Gee M.M. (2020). A Comparison of Methods for the Isolation and Separation of Extracellular Vesicles from Protein and Lipid Particles in Human Serum. Sci. Rep..

